# Ferulic Acid Alleviates Intestinal Inflammatory Damage in Mice, Associated with Ameliorating Intestinal Barrier Damage and Gut Microbiota

**DOI:** 10.3390/ani15182698

**Published:** 2025-09-15

**Authors:** Huan Huang, Jiehang Li, Jingru Zuo, Yanhua Qiu, Bing Li, Rongbin Hu, Yubin Bai, Jiyu Zhang

**Affiliations:** 1 Key Laboratory of New Animal Drug Project of Gansu Province, Lanzhou 730050, China; 2 Key Laboratory of Veterinary Pharmaceutical Development, Ministry of Agriculture, Lanzhou 730050, China; 3 Lanzhou Institute of Husbandry and Pharmaceutical Sciences, Chinese Academy of Agricultural Sciences, No.335 Jiangouyan, Xihu Street, Lanzhou 730050, China

**Keywords:** ferulic acid, intestinal barrier, gut microbiota, intestinal inflammatory damage, lipopolysaccharide

## Abstract

Intestinal inflammation remains one of the most common and severely harmful diseases in intensive farming systems. Ferulic acid (FA) has significant anti-inflammatory properties, but its role in intestinal inflammatory damage remains unclear. The aim of this study is to investigate the protective effect of FA (20, 40, 80 mg/kg) on lipopolysaccharide (LPS)-induced intestinal inflammatory damage in mice and its possible mechanism. FA treatment significantly reduced serum DAO and D-LA levels and suppressed intestinal expression of IL-1β, IL-6, and TNF-α. FA enhanced intestinal antioxidant activity by increasing GSH, SOD, and CAT levels, while reducing MDA concentrations. FA can increase the content of MUC-2 and promote the secretion of sIgA. The results of 16s rRNA gene sequencing revealed that FA regulated gut microbiota disorders associated with inflammation and mucosal barrier. The present study demonstrates that FA can alleviate LPS-induced intestinal inflammatory damage in mice by inhibiting inflammation, enhancing intestinal barrier function, and regulating the intestinal flora balance.

## Simple Summary

Gram-negative bacterial infections cause significant economic losses in livestock and poultry farming due to enteritis. Ferulic acid (FA) is a natural phenolic compound with excellent anti-inflammatory properties and antimicrobial properties. This study investigated the effects of FA on lipopolysaccharide (LPS)-induced acute intestinal inflammatory damage in animals, using an LPS-induced acute intestinal inflammation damage model. The results showed that, compared to the model group, FA significantly reduced intestinal inflammatory and oxidative factors induced by LPS, while promoting the expression of antioxidant factors and barrier-related proteins. Additionally, it exhibited remarkable regulatory effects on the gut microbiota.

## 1. Introduction

High-density livestock farming increases the incidence of intestinal diseases like post-weaning diarrhea and enteritis, leading to poor growth, feed inefficiency, and high mortality, causing serious economic losses [1]. Moreover, the incidence of intestinal inflammatory damage in livestock and poultry is increasing year by year, seriously threatening the lives and health of these animals. Intestinal inflammatory damage is a disease mainly characterized by damage to the intestinal mucosal barrier and inflammatory response [2]. Intestinal inflammatory diseases refer to a series of disorders that cause excessive inflammatory responses in the animal’s intestines, damage to the intestinal barrier function, and imbalance of the gut microbiota [3]. Its clinical manifestations include diarrhea, hematochezia, vomiting, pain, etc. [4]. During the development of intestinal inflammatory damage, impaired intestinal barrier function may lead to abnormal physiological and biochemical indexes of the body [5]. The intestinal tract provides a habitat for gut microbes. The intestinal microecosystem has been proven to be a key regulator influencing the health status of the host [6]. Relevant studies have shown that the imbalance of the gut microbiota can lead to the leakage of endotoxins and trigger systemic inflammation [7]; it is manifested as an abnormal increase of pro-inflammatory cytokines (IL-1β, IL-6, TNF-α) and an increase in intestinal permeability, which aggravates damage to the intestinal barrier [8]. Therefore, intestinal barrier repair combined with flora regulation are strategies to combat intestinal inflammatory damage. Although antibiotics are commonly used in clinical treatment and have good effects, when the intestinal microbiota is exposed to antibiotics, it may lead to intestinal inflammatory responses and the occurrence of other diseases [9]. Natural compounds have been reported to alleviate intestinal inflammation by restoring gut microbiota composition, improving epithelial barrier function, and suppressing pro-inflammatory cytokine expression [10].

As a natural phenolic substance, ferulic acid (FA) is abundantly available and can be found in various foods in our daily diet, such as plants (rice, wheat, oats), vegetables (lettuce), fruits (pineapple, citrus, grapes) [11]. FA has anti-inflammatory, antioxidant, antibacterial, anti-tumor, and other biological activities [12]. These properties make FA one of the most promising candidates for treating diseases in which inflammation and oxidative stress are key causative factors [13]. In recent years, the role of FA in intestinal health has gradually gained attention. For example, FA can alleviate arsenic-induced intestinal damage by regulating the expression level of intestinal epithelial tight junctions and inhibiting oxidative stress [14]. In addition to its direct anti-inflammatory effects, FA has demonstrated capacity to modulate gut microbiota composition in metabolic disease models, enhancing microbial diversity and promoting beneficial bacterial taxa [15]. At present, the research on the intestinal protective effect of ferulic acid (FA) in the intestinal inflammation injury model induced by lipopolysaccharide (LPS) and its potential molecular mechanisms still requires further in-depth exploration.

This study utilized an LPS-induced murine model of intestinal inflammatory damage to comprehensively assess the therapeutic efficacy of FA at various concentrations, with particular focus on its modulatory effects on intestinal inflammatory responses, epithelial barrier integrity, and gut microbiota composition. This study aims to provide substantial preclinical evidence supporting FA’s potential as a novel phytotherapeutic agent for managing intestinal inflammation.

## 2. Materials and Methods

### 2.1. Animal and Experimental Design

Six-week-old male SPF-grade BABL/c mice were purchased from the Laboratory Animal Center of Lanzhou Veterinary Research Institute of Chinese Academy of Agricultural Sciences. They were housed at 25 °C on a 12 h light/dark cycle, fed and watered ad libitum. Fifty SPF mice were randomly divided into five experimental groups (*n* = 10), i.e., the control group (control), model group (LPS), FA high-dose group (H-FA, 80 mg/kg) (HY-N0060, MCE, South Brunswick Township, NJ, USA), FA medium-dose group (M-FA, 40 mg/kg), and FA low-dose group (L-FA, 20 mg/kg). All the FA-dose groups received intragastric administration of different doses of the drug for 7 consecutive days, while the control group and the model group were given saline. After administration, LPS group and all the FA groups of mice were intraperitoneally injected lipopolysaccharide (15 mg/kg) (L2880, Sigma, St. Louis, MO, USA) to induce intestinal inflammatory damage, and the control group was given the same amount of normal saline. At the end of the experiment, we performed euthanasia through cervical dislocation. This experiment was approved by the Experimental Animal Ethics Review Committee of the Lanzhou Institute of Husbandry and Pharmaceutical Sciences (Permit No. 2024-42).

### 2.2. Body Weight and Organ Index

At the end of the experiment, the heart, kidney, lung, spleen, thymus, and liver tissues of mice were extracted immediately to calculate the organ index. The calculation formula was as follows: Organ Index = Organ weight (mg)/Body weight (g).

### 2.3. Serum D-LA and DAO Were Detected in Mice

Blood was collected from mice and serum was isolated. D-lactic acid (D-LA) content and diamine oxidase (DAO) activity in serum were detected by kit (JL-T2401, JL-T0649, Jonlnbio, Shanghai, China). For D-LA determination, according to the instructions of the kit, the corresponding reagent and sample were added separately, mixed well, and incubated at 37 °C for 30 min. The absorbance at 450 nm was read. The difference between the test sample and the blank tube was calculated and substituted into the formula for calculation. For DAO determination, according to the instructions of the kit, the corresponding reagents 1–4 were added, respectively, mixed well, and incubated at 30 °C for 5 min. Then, reagent 5 was added and mixed well. The absorbance at 340 nm was read after 30 °C incubation. The absorbance was measured again after 30 min, and the difference was calculated and substituted into the formula for calculation.

### 2.4. Histological Analysis

The jejunal tissue was fixed with 4% paraformaldehyde for one night. The intestinal tissue was trimmed, then embedded in paraffin. The paraffin blocks were trimmed and cut into 4 μm sections. These sections were stained with HE (G1076, Servicebio, Wuhan, China) and observed under a microscope.

### 2.5. Enzyme-Linked Immunosorbent Assay (ELISA)

TNF-α, IL-1β, IL-6, MUC-2, and sIgA in the intestinal tissue of mice were determined according to the instructions of ELISA kit (JL20268, JL18442, JL10484, JL47609, JL11763, Jonlnbio, Shanghai, China). The supernatant of the homogenate was added to the corresponding wells, then incubated. The required working solution for the reaction was added as per the instructions of the kit, and finally, the stop solution was added. Measurements of the OD value of each well were taken at 450 nm.

### 2.6. Determination of Intestinal NO Content

The content of NO (S0021S, Beyotime, Haimen, China) in the intestinal tract of mice was detected using an NO kit, following the instructions of the test kit for operation. A standard curve was prepared according to the requirements of the test kit. The measured values were input into the standard curve to calculate the content of NO.

### 2.7. Determination of Intestinal Oxidative Stress Related Indexes

The contents of glutathione (GSH) (BC1175, Solarbio, Beijing, China), superoxide dismutase (SOD) (BC5165, Solarbio, Beijing, China) activity, catalase (CAT) (BC0205, Solarbio, Beijing, China) activity, and malondialdehyde (MDA) (BC0025, Solarbio, Beijing, China) in the intestinal tissues of mice were detected according to the instructions of the kit. The instructions were followed to perform sample addition and other operations, and the measured absorbance was substituted into the formula for calculation.

### 2.8. Real-Time Reverse Transcription-Polymerase Chain Reaction (RT-qPCR)

Total RNA was extracted and its concentration was measured. It was then reverse-transcribed into cDNA. And the mRNA expression levels of inflammation-related factors and tight junction proteins were detected by fluorescence quantitative PCR. *β-actin* was used as an internal reference gene, and the relative expression levels of mRNA of each target gene in each group were calculated using the 2^−ΔΔ*Ct*^ method. The primer sequences utilized in this study are detailed in Table 1.

### 2.9. Gut Microbiota 16S rRNA Sequencing

The total DNA of mouse cecum contents was extracted, the molecular size was determined by 0.8% agarose gel electrophoresis, and the DNA was quantified using Nanodrop. And the highly variable V3-V4 region of bacterial 16S rRNA gene was amplified by PCR using universal primes 338F (5′-CCTAYGGGRBGCASCAG-3′) and 806R (5′-GGACTACNNGGGTATCTAAT-3′). Finally, sequencing was performed on the Illumina MiSeq platform.

### 2.10. Statistical Analysis

The experimental data were analyzed using IBM SPSS Statistics 26.0 software (International Business Machines Co., Ltd., Armonk, NY, USA) and results are presented in the form of mean ± standard deviation (SD). Gut microbiota sequencing data were evaluated using the Kruskal–Wallis test. The statistical significance threshold was set at *p* < 0.05.

## 3. Results

### 3.1. Protective Effect of FA on LPS-Induced Intestinal Inflammatory Damage in Mice

The data demonstrated that body weight increased progressively in all mouse groups over the administration period (Table 2), which indicated that the different dosages of FA did not cause slow growth, loss of appetite, or emaciation of mice, and the mice were in a normal state of growth. As shown in Table 3, the organ indices of mice after LPS treatment all showed a significant increase. Compared with the LPS group, FA significantly decreased the liver index of mice (*p* < 0.05), and had a tendency to decrease the heart, lung, spleen, kidney, and thymus index of mice.

### 3.2. FA Alleviates Intestinal Tissue Damage in Mice with Intestinal Inflammatory Damage

**Figure 1:**
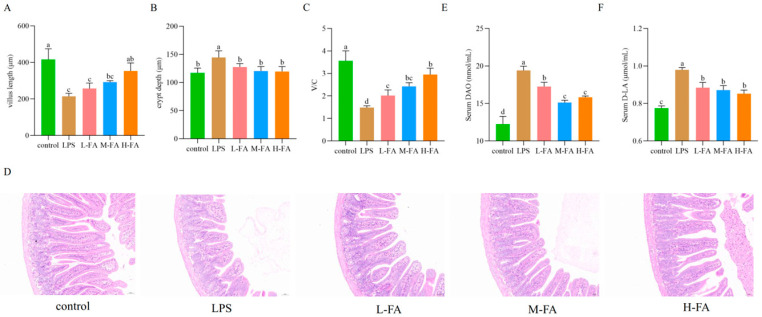
Intestinal morphology and intestinal permeability in mice: (**A**) Villus length; (**B**) Crypt depth; (**C**) V/C; (**D**) Intestinal HE staining; (**E**) DAO; (**F**) D-LA. (n = 3). Note: Value columns with the same small letter mean no significant difference (*p* > 0.05), while different small letters mean significant difference (*p* < 0.05).

As shown in the figure below, the intestinal villus of mice in the control group were intact. Compared with the control group, villus in the LPS group were atrophied, their length was significantly reduced, and the ratio of villus length to crypt depth was significantly decreased. Compared with the LPS group, the villus structure of FA group was restored to a certain extent, villus length was significantly increased, and the ratio of villus length to crypt depth (V/C) was also significantly increased (*p* < 0.05) (Figure 1A–D). The intestinal permeability of mice could be evaluated by detecting the content of D-lactic acid (D-LA) content and diamine oxidase (DAO) in serum. The results showed that after LPS injection, the contents of DAO and D-LA in the serum of mice increased significantly (*p* < 0.05), which means the intestinal permeability of mice was increased. However, different concentrations of FA doses significantly reduced the levels of DAO and D-LA in the serum of the mice (*p* < 0.05), that is, the intestinal permeability decreased (Figure 1E,F).

### 3.3. FA Improved the Level of Intestinal Inflammatory Factors in Mice with Intestinal Inflammatory Damage

**Figure 2:**
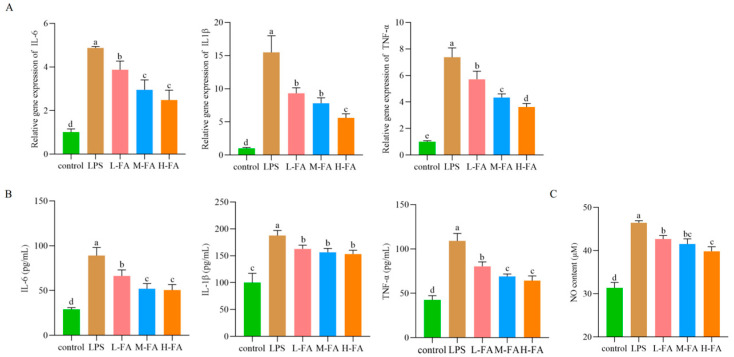
Inflammatory factors in the mouse gut: (**A**) Gene expression levels (n = 4); (**B**) Protein expression levels; (**C**) NO content. (n = 3). Note: Value columns with the same small letter mean no significant difference (*p* > 0.05), while different small letters mean significant difference (*p* < 0.05).

The response of FA to inflammation was evaluated by measuring pro-inflammatory cytokines in intestinal tissues after LPS stimulation. As shown in Figure 2A, mRNA levels of IL-6, IL-1β, and TNF-α were significantly up-regulated after intraperitoneal injection LPS compared with the control group (*p* < 0.05). Compared with LPS group, FA group significantly down-regulated the mRNA levels of IL-6, TNF-α, and IL-1β in intestinal tissues (*p* < 0.05). After LPS injection, the protein expression levels of intestinal inflammatory factors were consistent with the gene levels. However, FA at different doses significantly reduced the content of intestinal proinflammatory factors (*p* < 0.05) (Figure 2B). Quantifying the level of the pro-inflammatory mediator NO is also an indicator for evaluating inflammation. Compared with control group, LPS significantly increased the intestinal NO content of mice (*p* < 0.05). However, FA significantly reduced the intestinal NO content in a dose-dependent manner (*p* < 0.05) (Figure 2C). These results suggest that different doses of FA can improve the intestinal inflammatory response induced by LPS in mice.

### 3.4. FA Improves Intestinal Oxidative Stress in Mice with Intestinal Inflammatory Damage

**Figure 3:**
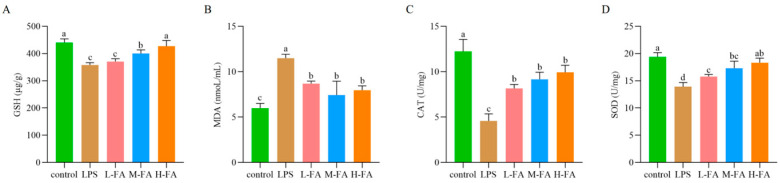
Determination of oxidative-related indicators in the mouse intestine: (**A**) GSH; (**B**) MDA; (**C**) CAT; (**D**) SOD. (n = 3). Note: Value columns with the same small letter mean no significant difference (*p* > 0.05), while different small letters mean significant difference (*p* < 0.05).

As shown in the Figure 3, compared with the control group, LPS significantly decreased the intestinal GSH content, CAT activity, and SOD activity of mice, and significantly increased the MDA content (*p* < 0.05). Compared with the LPS group, FA alleviated the increase of intestinal oxidation factor content and promoted the increase of antioxidant factor content.

### 3.5. FA Improves Intestinal Mucosal Barrier in Mice with Enteritis

**Figure 4:**
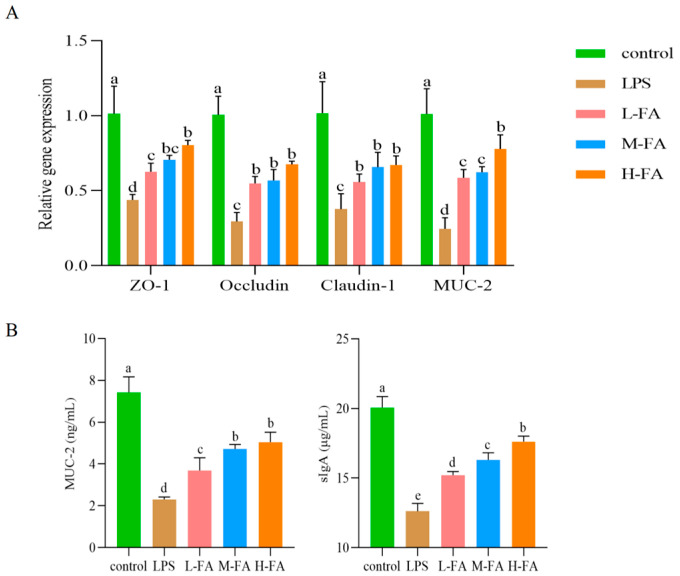
Gene and protein expression levels of intestinal mucosal barrier related factors in mice: (**A**) Gene expression levels (n = 4); (**B**) Protein expression levels (n = 3). Note: Value columns with the same small letter mean no significant difference (*p* > 0.05), while different small letters mean significant difference (*p* < 0.05).

Compared with the control group, LPS significantly down-regulated mRNA expression levels of zonula occludens-1 (ZO-1), occludin, claudin-1, and mucin 2 (MUC-2) in the mouse intestinal barrier (*p* < 0.05), indicating that the intestinal physical barrier and chemical barrier were damaged. Compared with the LPS group, FA alleviated the damage of intestinal physical barrier and chemical barrier caused by LPS in a dose-dependent manner, and significantly up-regulated the mRNA expression of ZO-1, occludin, claudin-1, and MUC-2 in the intestinal tract of mice (*p* < 0.05) (Figure 4A). The ELISA results were consistent with the MUC-2 gene level test. The secretion of sIgA was significantly decreased after injection of LPS; however, FA alleviated the decrease in sIgA secretion (*p* < 0.05) (Figure 4B).

### 3.6. FA Regulates Gut Microbiota in Mice with Enteritis

Numerous studies have shown a strong relationship between gut microbial imbalance and intestinal inflammation. We performed a comprehensive analysis using 16S rRNA gene sequencing. Through the previous experiments we concluded that the H-FA group had the best effect, therefore, we chose the H-FA group for 16S rRNA gene sequencing.

#### 3.6.1. Alpha and Beta Diversity Analysis of Gut Microbitoa

**Figure 5:**
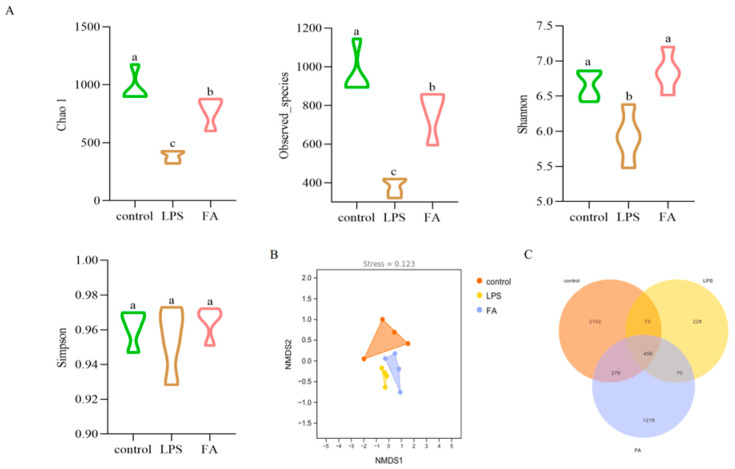
Diversity analysis: (**A**) Index of Alpha diversity; (**B**) Beta diversity analysis; (**C**) OTUs (n = 4). Note: Value columns with the same small letter mean no significant difference (*p* > 0.05), while different small letters mean significant difference (*p* < 0.05).

The Chao1, Shannon, Simpson, and observed species indices are important indicators to evaluate the species diversity of ecosystems. As shown in Figure 5A, compared with the control group, the Chao1, Shannon, and observed species indices of the LPS group decreased significantly, while the FA group’s observed species indices increased significantly (*p* < 0.05). These results suggest that FA can effectively alleviate the decrease of cecal microflora richness and diversity induced by LPS in mice.

The control group were completely separated from the LPS group, indicating that there were significant differences in community structure between the control group and the model group. LPS had a significant effect on the intestinal microflora of mice with induced enteritis, whereas the FA group was closer to the control group. This suggests that FA can improve the LPS-induced structural changes in the gut microbiota of the cecum in mice (Figure 5B).

As can be seen from the figure, the total number of gut microbiota OTUs in each group was 4428. The number of OTUs in the LPS group was lower than in the control group and FA group. The total number of OTUs in the control group was 2912, and the number of unique OTUs was 2102. The number of OTUs in LPS group was 829, and the number of unique OTUs was 228. The total number of OTUs in FA group was 2025, and the number of unique OTUs was 1218. This showed that LPS had a certain effect on the number of gut microbiota in mice (Figure 5C).

#### 3.6.2. Gut Microbiota Composition

**Figure 6:**
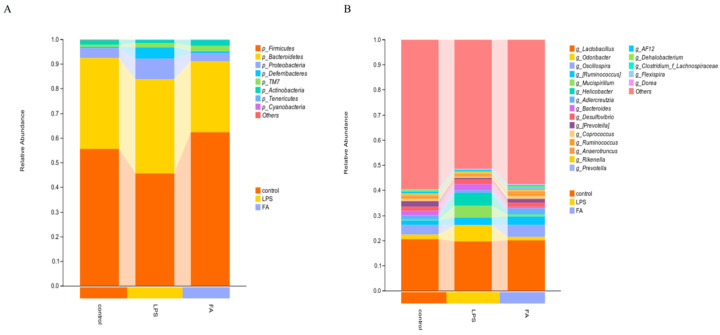
Gut microbiota composition. (**A**) Phylum level; (**B**) Genus level. (n = 4).

We further analyzed the differences in microbial community composition. At the phylum level (Figure 6A), Firmicutes in the LPS group decreased, while the abundance of *Bacteroidetes*, *Proteobacteria*, and *Deferribacteres* increased. FA alleviated the changes in gut microbiota composition induced by LPS. At the genus level (Figure 6B), *Odoribacter*, *Helicobacter*, *Mucispirillum*, and *Bacteroides* in the LPS group increased. *Oscillospira* in the LPS group decreased. These results indicate that LPS caused gut microbiota disorder in mice, while FA could regulate the transformation of gut microbiota compared to the control group.

#### 3.6.3. Statistical Analysis of Gut Microbiota Diversity and Predictive Analysis of Potential Functions

**Figure 7:**
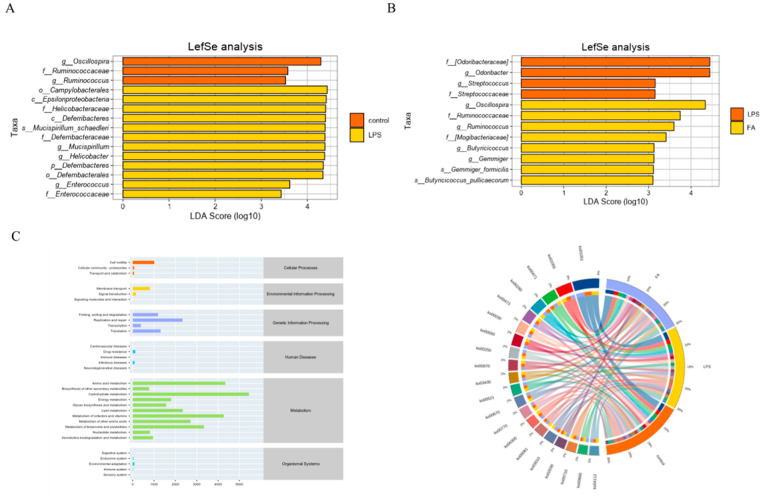
Multivariate statistical analysis and potential function prediction: (**A**) The results of LEFSe analysis between LPS and control group; (**B**) The results of LEFSe analysis of between LPS and FA group; (**C**) Potential function prediction (n = 4).

LEfSe is an analytical tool that can find and interpret high-latitude biological markers between two groups. As shown in Figure 7A, there were obvious differences in the composition of the microbiota between the two groups (a total of 15 different species had significant differences) the control group showed significant enrichment in three different species. The LPS group demonstrated significant advantages in 12 different species, including pathogenic bacteria such as *Helicobacter*, etc. As shown in Figure 7B, LPS group and FA group had significant differences at 12 different species, of which 4 were significantly enriched in the LPS group and 8 in the FA group (*p* < 0.05). In the FA group, bacteria such as *Oscillospira*, *Ruminococcus,* etc. contributed significantly to the inter-group differences.

According to PICRUSt-KEGG enrichment analysis, changes in the diversity and richness of gut microbiota in the cecum of mice also change the function of their gut microbiota. These include cellular processes, genetic information processing, and metabolism. The specific pathways involve carbohydrate metabolism, amino acid metabolism, cell motility, membrane transport, replication and repair, metabolism of cofactors and vitamins, and so on (Figure 7C).

### 3.7. Correlation of Gut Microbiota (At the Genus Level) with Intestinal Inflammation, Oxidation Index and Barrier-Related Factors

**Figure 8:**
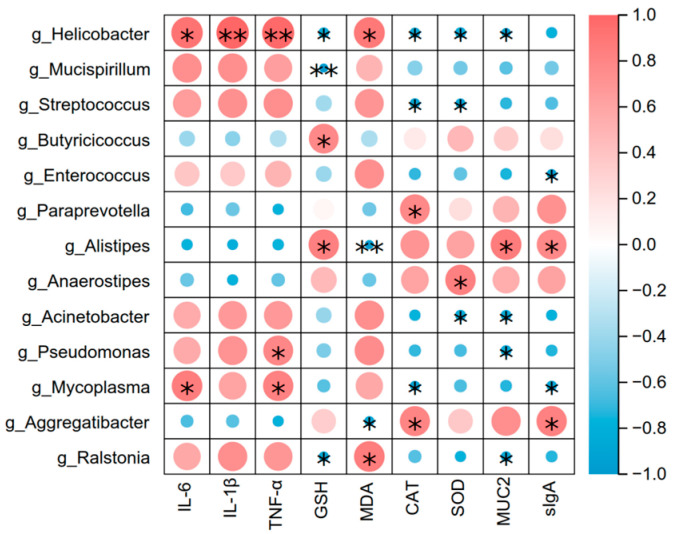
Correlation analysis of intestinal inflammatory factors, oxidative factors, barrier-related factors, and gut microbiota. “*” indicates significant correlation (*p* < 0.05), and “**” indicates extremely significant correlation (*p* < 0.01).

This study employed Pearson correlation analysis to systematically evaluate the potential associations between inflammatory factors, oxidative stress indicators, intestinal barrier-related factors, and the intestinal microbiota in a mouse model, aiming to reveal the underlying molecular mechanisms of host-microbiota interactions. As shown in Figure 8, *Helicobacter*, *Mucispirillum*, etc. were positively correlated with pro-inflammatory factors IL-6, IL-1β, TNF-α, and lipid peroxidation indicator MDA; *Helicobacter*, *Mucispirillum*, etc. were negatively correlated with antioxidant indicators GSH, CAT, SOD, and barrier-related factors. *Butyricicoccus*, *Alistipes,* etc. were positively correlated with antioxidant factors MUC2 and sIgA, and these were significantly enriched in the FA group. These indications suggest that bacterial groups such as *Helicobacter* and *Mucispirillum* may be associated with intestinal inflammatory damage and contribute to the progression of the disease.

## 4. Discussion

In recent years, with the rapid development of intensive livestock farming, the issue of intestinal inflammation in livestock and poultry has become increasingly prominent and has become one of the key factors restricting the sustainable development of the livestock industry. Chemical drugs such as antibiotics have played a significant role in the prevention and treatment of such diseases. However, with the long-term and excessive use of antibiotics, their drawbacks have become increasingly prominent, including issues such as the resistance of pathogenic microorganisms and drug residues. Therefore, the development of safe and effective natural products for the prevention and treatment of intestinal inflammatory damage holds significant clinical importance. FA is known for its anti-inflammatory, antioxidant, and antibacterial properties. For example, studies have shown that FA has a favorable therapeutic effect on ulcerative colitis [16], and its mechanism of action may be to attenuate inflammation, oxidation, and apoptosis in ulcerative colitis by inhibiting the NF-κB signaling pathways [17]. Research on the role and mechanism of FA in alleviating intestinal inflammatory damage is still relatively limited. LPS is the main component of the outer membrane of Gram-negative bacteria and has a strong immune-stimulating activity. LPS also has a wide range of toxic effects, it is easy to control, and it offers good reproducibility; so, it is often used as an inducer for intestinal injury and inflammation models [18,19]. We referred to the research of Zhang et al. and selected a dose of 15 mg/kg (body weight) to induce the intestinal inflammatory injury model [20].

Intestinal inflammatory damage usually results in diarrhea and weight loss [18]. We induced symptoms such as diarrhea, weight loss, and intestinal tissue injury in the mouse enteritis model by intraperitoneal injection of LPS. The symptoms are consistent with the typical clinical manifestations of intestinal inflammation models [21]. The microscopic observation of intestinal tissues showed that the animal intestinal injury model was successfully established. From the general viewpoint of the experimental process, different doses of FA played a role in the improvement of body weight maintenance and mental status of mice after LPS treatment. The organ index reflects the state of health of an organism. With organ injury caused by inflammation, the organ indexincreases [22]. In this study, different doses of FA were found to significantly alleviate the LPS-induced increase in liver official index, suggesting that FA may improve the clinical symptoms of mice by alleviating LPS-induced liver injury. The V/C is related to intestinal nutrient intake and is also an important indicator of intestinal health [23]. Many studies have shown that LPS leads to a significant shortening of the length of the small intestinal villus [24,25]. The results of this study are consistent with Wang’s [14] findings that FA improves intestinal morphology in enteritis.

Chemical markers (DAO, D-LA) are important indicators of intestinal permeability [26]. The increase in intestinal mucosal permeability indicates that the intestinal physical barrier function has been impaired, resulting in the release of a large amount of D-LA into the blood. DAO exists in intestinal mucosa and ciliary cells. When the intestinal barrier is damaged, a large amount of highly active intracellular enzyme DAO released by intestinal mucosal epithelial cells will enter the blood circulation, resulting in increased blood DAO content [27]. The results of this study, as well as Tang [28], both indicate that FA reduces intestinal permeability. Increased intestinal permeability allows LPS produced by intestinal bacteria to diffuse into the bloodstream, a process that not only triggers bacterial translocation but also activates the inflammatory response, resulting in increased release of inflammatory cytokines [29]. IL-6, IL-1β, and TNF-α are considered biomarkers of inflammation and are positively correlated with the severity of enteritis [30]. Wang’s [12] research results show that FA can reduce the expression of inflammatory factors. The results of this study once again confirm that FA has a very good inhibitory effect on intestinal inflammatory factors. Specifically, we show the mRNA and protein expression levels of IL-6, IL-1β, and TNF-α genes in the intestinal tract of mice were significantly increased after induction by LPS, while FA could alleviate the increase of inflammatory factors. NO is closely related to the occurrence and development of inflammatory diseases and is a landmark product in inflammatory response. It is regulated by inducible nitric oxide synthase (iNOS). When stimulated by LPS, iNOS catalyzes the production of a large amount of NO, promoting inflammation and tissue damage [31]. The experimental data showed that the intestinal NO content of mice in the LPS stimulation group was significantly increased compared with the control group. The change of this biochemical index was closely related to the pathological process of enteritis, verifying the successful construction of the mouse enteritis model induced by LPS. FA decreased the release of NO in the intestinal tract of LPS-induced enteritis mice in a dose-dependent manner. This is consistent with the conclusion that FA has excellent anti-inflammatory effect reported in Mahsa Ekhtiar’s studies [16]. These results suggest that FA can reduce intestinal inflammatory response and improve intestinal inflammation in mice with LPS-induced intestinal inflammatory damage.

Oxidative stress is a concomitant reaction highly associated with inflammation, and is also one of the indicators to judge the development of inflammatory bowel disease [32]. During inflammation, the body is prone to an imbalance in the oxidative-antioxidant system [33]. MDA is the final decomposition product of membrane lipid peroxidation, which can reflect the rate and intensity of lipid peroxidation. Antioxidant enzymes GSH, SOD, and CAT are important components of the antioxidant system, which can reflect the antioxidant capacity of the body as a whole [34]. Therefore, this study evaluated the antioxidant capacity of FA by detecting intestinal oxidative stress levels. In this study, MDA, as the signature product of lipid oxidation [35] was significantly increased after LPS injection, while GSH, CAT, and SOD antioxidant enzymes were significantly decreased, which may have been due to ROS overload caused by intestinal injury, leading to the body entering the stage of oxidative damage. This is consistent with Zhang’s [36] findings. However, FA was able to decrease the increase of MDA and increase the contents of GSH, CAT, and SOD. These antioxidant effects of FA corroborate earlier reports [14] where dietary interventions successfully mitigated oxidative stress markers and restored antioxidant enzyme activity in gut injury models.

Restoration of tight junction proteins and mucin production is a critical component of anti-inflammatory interventions in intestinal disorders, a strategy also adopted by drugs in treating related disease models [37]. Tight junction proteins can regulate intestinal permeability and maintain the integrity of intestinal barrier, and ZO-1, occludin, and claudin-1 are important among its components [38]. Mucins are an important part of the intestinal chemical barrier secreted by goblet cells into the intestinal lumen [39]. In the intestinal cavity, sIgA is the primary effector in intestinal mucosal immunity, which can protect the intestinal epithelium from pathogens and toxins and act as the first line of defense of the immune barrier [40]. The results of this study indicate that after intraperitoneal injection of LPS, the mRNA expression levels of intestinal ZO-1, occludin, claudin-1, MUC-2, and the secretion of sIgA were affected; FA restored the expression levels of intestinal barrier-related factors in a dose-dependent manner. These suggest that FA may alleviate the intestinal inflammation damage caused by LPS in mice by improving the intestinal barrier. Wang’s research indicates that FA may maintain the integrity of the intestinal barrier and inhibit oxidative stress by suppressing the activation of the NFκB and Nrf2/HO-1 signaling pathways. In the subsequent stage of this research, we will further explore the mechanism of action of FA based on the existing reports.

An imbalance of the gut microbiota can lead to immune dysfunction and promote inflammation [41]. High abundance and diversity of the gut microbiota are positively correlated with intestinal stability. Alpha diversity is often closely related to intestinal diseases [42]. This study found that LPS caused a significant decline in Chao1, Shannon, and observed species indices in the gut microbiota of mice. Similar reduction in microbial diversity has been similarly reported in toxin-induced intestinal injury models, where disruptions in cecal microbial richness were associated with pro-inflammatory metabolite accumulation and impaired epithelial barrier repair [43]. FA was able to restore the richness and diversity of gut microbiota caused by LPS. This is consistent with Tang’s conclusion that FA has a regulatory effect on the intestinal microbiota of Tianfu broilers induced by LPS [28]. Previous studies have reported that LPS has ability to destroy the fragile structure of gut microbiota, thus triggering it imbalance [44]. At the phylum level, Bacteroidetes and *Firmicutes* dominated the gut microbiota in all populations, and the results of this study showed that these two phyla were the dominant phyla, in line with normal animal intestinal phyla [45]. The dynamic balance of Bacteroidetes and thick-walled bacteria is an important basis for ensuring the physiological function of the intestinal tract [46]. In this study, Firmicutes decreased, Bacteroidetes, Proteobacteria, and *Deferribacteres* increased in the mice enteritis model induced by LPS. These were consistent with Chen’s results [47]. From the perspective of microbiological characteristics, Proteobacteria, as a typical Gram-negative bacterial community, contains a large amount of LPS with strong anti-inflammatory activity in its cell wall structure. Notably, excessive proliferation of *Proteobacteria* and *Deferribacteres* not only elevates endotoxin burden but also exacerbates intestinal mucosal injury [48]. Further observation showed that LPS increased the abundance of *Bacteroides* in class, family and genus, while FA decreased the abundance of *Bacteroides* in different levels [49]. Further study showed that *Helicobacter* and *Mucispirillum* were enriched after LPS treatment, and similar phenomenon was also observed in this study. By LEfSe analysis, LPS-induced intestinal inflammation in mice identified *Helicobacter*, *Helicobacteraceae*, *Deferribacteres*, *Deferribacterales*, and other biomarkers. These bacteria are common pathogenic bacteria of intestinal diseases [50]. We found that the enrichment of pathogens such as *Helicobacter* and *Defferibacteres* was not identified in FA, which was consistent with the results of Dai [51]. The current findings strongly suggest that FA may reshape the gut microbiota in the cecum of mice to alleviate the intestinal inflammatory damage induced by LPS.

## 5. Conclusions

In this study, we investigated the effect of FA on LPS-induced intestinal inflammatory damage in mice. The results show that FA can reduce the clinical symptoms of intestinal inflammatory damage in mice, enhance their intestinal barrier function, alleviate the inflammatory response, oxidative stress, and intestinal permeability induced by LPS in mice, and it also has a good regulatory effect on gut microbiota. In summary, this study provides theoretical support for FA as a natural medicine in preventing or regulating the symptoms of intestinal inflammatory damage. In the future, it is necessary to further explore the mechanism of action between inflammatory factors and gut microbiota. Beyond restoring microbial balance, FA treatment may also indirectly modulate gut microbiota-derived metabolites and hepatic pathways. Studies should also investigate the impact of FA on gut microbial metabolite outputs, to provide better understanding of FA’s therapeutic impact.

## Figures and Tables

**Table 1:** Primer sequences.

Gene	Accession Number	Primer	Size (Base)
*β-actin*	NM_007393.5	F: ACTGCCGCATCCTCTTCCTC R: AACCGCTCGTTGCCAATAGTG	80
*IL-6*	NM_001314054.1	F: CGGAGAGGAGACTTCACAGAGG R: TTCCACGATTTCCCAGAGAACATG	102
*IL-1β*	NM_008361.4	F: TCGCAGCAGCACATCAACAAG R: TCCACGGGAAAGACACAGGTAG	94
*TNF-α*	NM_001278601.1	F: ACGTGGAACTGGCAGAAGAGG R: TGAGAAGAGGCTGAGACATAGGC	86
*ZO-1*	NM_001163574.2	F: AGGAGGTAGAACGAGGCATCATC R: CCCGCTGTCTTTGGAAGTGTG	85
*Occludin*	NM_001360536.1	F: GGCGGCTATGGAGGCTATGG R: CTAAGGAAGCGATGAAGCAGAAGG	106
*Claudin-1*	NM_016674.4	F: CCTGGCTTCTCTGGGATGGATC R: CTGAGCGGTCACGATGTTGTC	97
*MUC2*	NM_023566.4	F: GAGCACATCACCTACCACATCATC R: AATCCAGCCAGCCAGTCCAC	89

**Table 2:** Body weight of mice (g) (n = 10).

Days	1	2	3	4	5	6	7	#
control	20.79 ± 0.87	21.34 ± 0.81	22.23 ± 0.80	21.90 ± 1.30	22.16 ± 0.83	22.36 ± 1.05	22.57 ± 1.12	22.34 ± 1.15
LPS	20.80 ± 1.39	21.18 ± 1.18	21.49 ± 1.19	21.90 ± 1.17	22.09 ± 1.12	21.74 ± 1.06	21.94 ± 1.21	20.84 ± 1.11
L-FA	20.40 ± 1.05	20.98 ± 0.84	21.15 ± 1.39	21.28 ± 0.66	21.61 ± 0.82	21.93 ± 0.82	21.85 ± 0.77	20.98 ± 0.81
M-FA	20.92 ± 0.83	21.48 ± 0.92	21.96 ± 1.11	21.63 ± 0.85	21.82 ± 0.68	22.53 ± 0.84	22.79 ± 0.68	21.95 ± 0.50
H-FA	20.82 ± 1.51	20.99 ± 1.29	21.30 ± 0.67	21.35 ± 1.30	21.81 ± 1.13	21.91 ± 1.06	21.96 ± 0.69	21.20 ± 0.89

Note: “#” indicates 6 h after the injection of lipopolysaccharide or saline.

**Table 3:** Organ index (mg/g) (n = 10).

Organ Index	Heart Index	Liver Index	Lung Index	Spleen Index	Kidney Index	Thymus Index
control	5.91 ± 0.69b	44.47 ± 2.12c	3.24 ± 0.62b	6.64 ± 0.85b	13.57 ± 0.90b	1.63 ± 0.34b
LPS	7.18 ± 1.09a	52.21 ± 2.20a	5.37 ± 0.66a	7.62 ± 0.60a	15.48 ± 1.15a	2.06 ± 0.27a
L-FA	6.76 ± 0.83a	49.15 ± 1.34b	5.04 ± 0.39ab	7.02 ± 0.53a	15.39 ± 1.11a	1.73 ± 0.23ab
M-FA	6.89 ± 0.71a	48.39 ± 2.17b	4.79 ± 0.55ab	7.24 ± 0.42a	15.38 ± 0.90a	1.71 ± 0.27ab
H-FA	6.80 ± 0.71a	48.61 ± 2.03b	5.05 ± 0.45ab	7.08 ± 0.60a	15.51 ± 0.80a	1.78 ± 0.48ab

Note: Value columns with the same small letter mean no significant difference (*p* > 0.05), while different small letters mean significant difference (*p* < 0.05).

## Data Availability

Sequence data that support the findings of this study have been deposited in the NCBI with the accession ID PRJNA 1265926.
